# The Genome Sequence of the Jean-Talon Strain, an Archeological Beer Yeast from Québec, Reveals Traces of Adaptation to Specific Brewing Conditions

**DOI:** 10.1534/g3.120.401149

**Published:** 2020-06-30

**Authors:** Anna Fijarczyk, Mathieu Hénault, Souhir Marsit, Guillaume Charron, Tobias Fischborn, Luc Nicole-Labrie, Christian R. Landry

**Affiliations:** * Département de Biologie, Université Laval, Québec, G1V 0A6, Canada; † Institut de Biologie Intégrative et des Systèmes (IBIS), Université Laval, Québec, G1V 0A6, Canada; ‡ Département de Biochimie, Microbiologie et Bioinformatique, Université Laval, Québec, G1V 0A6, Canada; § PROTEO, Le réseau québécois de recherche sur la fonction, la structure et l’ingénierie des protéines, Université Laval, Québec, G1V 0A6, Canada; ** Centre de Recherche en Données Massives (CRDM), Université Laval, Québec, G1V 0A6, Canada; †† Lallemand Inc., Montreal, H1W 2N8 Québec, Canada; ‡‡ National Battlefields Commission, Quebec, G1R 2L3, Canada

**Keywords:** yeast, polyploidy, long-read sequencing, beer brewing, archeology

## Abstract

The genome sequences of archeological *Saccharomyces cerevisiae* isolates can reveal insights about the history of human baking, brewing and winemaking activities. A yeast strain called Jean-Talon was recently isolated from the vaults of the Intendant’s Palace of Nouvelle France on a historical site in Québec City. This site was occupied by breweries from the end of the 17^th^ century until the middle of the 20^th^ century when poisoning caused by cobalt added to the beer led to a shutdown of brewing activities. We sequenced the genome of the Jean-Talon strain and reanalyzed the genomes of hundreds of strains to determine how it relates to other domesticated and wild strains. The Jean-Talon strain is most closely related to industrial beer strains from the beer and bakery genetic groups from the United Kingdom and Belgium. It has numerous aneuploidies and Copy Number Variants (CNVs), including the main gene conferring cobalt resistance in yeast. The Jean-Talon strain has indeed higher tolerance to cobalt compared to other yeast strains, consistent with adaptation to the most recent brewing activities on the site. We conclude from this that the Jean-Talon strain most likely derives from recent brewing activities and not from the original breweries of Nouvelle France on the site.

The budding yeast *Saccharomyces cerevisiae* has a long history of domestication by humans for the production of fermented food and beverages (Marsit *et al.* 2017). Among the oldest evidence of this domestication are traces of DNA from more than 3000 years old wine jars in Egypt (Cavalieri *et al.* 2003). Signs of beer and chemical traces of beer production dating back from the last half of the fourth millennium before the common era were identified in Sumerian artifacts (Michel *et al.* 1992). Alcoholic beverages were also present in prehistoric China about 9000 years before present (McGovern *et al.* 2004). Human populations brought fermented produces with them during their migration, including wine (Marsit *et al.* 2017), coffee and cacao beans (Ludlow *et al.* 2016), and this human assisted migration has contributed to the genetic organization of today’s population structure of *S. cerevisiae* (Peter *et al.* 2018). For instance, the genome analysis of some ale beer strains recently revealed that they originated from both European wine strains and Asian rice wine strains (Fay *et al.* 2019).

The production of fermented beverages by European settlers in North America started early in the colonies, for instance in the North American territory that became Nouvelle France and eventually Québec. A common drink for French settlers in the 17^th^ century called “bouillon” was made of bread leaven that was incubated in water, producing a lightly alcoholic beverage. Artisanal and domestic beer brewing most likely started during that period (Moussette 1992). Later in the 17^th^ century, the first industrial brewery was founded by the French King representative, the Intendant Jean Talon (Moussette 1996).

To establish his brewery, Talon acquired a relatively large lot at the crossing of St. Charles and St. Lawrence rivers in Québec City, and built a massive 40 meters long building with modern brewing equipment (Moussette 1994). The brewery was short lived (1670-1675). However, after the British conquest (1763), entrepreneurs turned to brewing again. The lot with Talon’s old brewery had been turned into a powder storage and the Intendant’s Palace (1686-1713), which was destroyed by a fire and served as warehouses for the remainder of Nouvelle France.

In the early 19^th^ century, the site went back to the brewing industry, thanks to the most successful family in Québec City’s brewing business, the Boswells. Joseph Knight Boswell was an Irish migrant who had been trained in Scotland. He settled in Québec City in 1830 and quickly worked as a brewmaster before opening his own business, Anchor Brewery, in the 1840s. Boswell expanded his business throughout the 1840s and finally linked his booming brewery with Talon’s brewery in 1852 by renting the plot on which Talon’s brewery was built in the 17^th^ century (Guimont 1987). The French vaults that Boswell rented were not from Talon’s brewery, but from the Second Intendant’s Palace (1713-1725), built slightly north of the brewery’s original site. Boswell would first use the 18^th^ century vaults to store beer. Then, in 1875, he had a large building built on top of the vaults for his malting equipment and operations (Fiset 2001). In the 1930s the Brewery opened “Les Voûtes Talon”, a pub located in the historical vaults. Beer production came to an end after the events of 1966 when beer brewed in Québec City caused nearly 50 deaths among heavy drinkers over the course of a few weeks. Public health authorities linked this to the use of cobalt sulfate as a stabilizer in the brewing process (Morin and Daniel 1967). The public relations disaster killed the brand brewed in Québec City (then labeled “Dow”) and led to the complete shutdown, in 1968, of the facility located on the same lot as Jean Talon’s brewery, ending a nearly 300 years long history.

Here, we sequenced the genome of a yeast strain (Jean-Talon) that was isolated from the Second Intendant’s Palace’s vaults. We report the whole-genome sequence using short- and long-read sequencing and the comparative analysis of this genome with other sequenced genomes. Our results reveal that the strain is polyploid, partially sterile and harbors multiple aneuploidies and copy number variants. A phylogenetic analysis reveals that it is indeed a beer strain that has recently diverged from other industrial beer and baking strains. A growth assay shows that the Jean-Talon strain is more tolerant to cobalt than other beer, wild or laboratory strains, suggesting it could have adapted to cobalt-rich brewing conditions present in the recent brewery that existed on the site, supporting the fact that it originated from more recent industrial activities and not from the original brewery of Nouvelle France.

## Materials and Methods

### Strain sampling

30 Yeast Mold Agar (YM) plates (Difco 271210) with 25 ppm of chloramphenicol to prevent bacterial growth were prepared. Sets of three YM plates were placed at 10 different locations in the vaults of the Second Intendant’s Palace of Nouvelle France in September 2010. At each location, the three YM plates were exposed to the environment for 10, 20 and 40 min and then incubated at 28° for two days. The yeast strain was banked at the Siebel culture collection as BRY # 480 and sent to the Landry laboratory for analysis in 2019.

### DNA content and ploidy

Measurement of DNA content was performed using flow cytometry and the SYTOX green staining assay (Thermo Fisher, Waltham, USA) as done in (Charron *et al.* 2019). Cells were first thawed from glycerol stock and streaked on solid YPD in six petri dishes (room temperature, three days) to obtain isolated colonies. The strain BY4742 (haploid) and MG009 (BY4741xBY4742) (diploid) were used as controls. Liquid YPD cultures of 1 ml from 90 Jean-Talon isolated colonies and the two controls in 96-deepwell (2 ml) plates were inoculated and incubated for 24 h at room temperature. Multiple colonies were considered to account for the possibility of an unstable ploidy. Cells were subsequently prepared as in (Gerstein *et al.* 2006). Cells were first fixed in 70% ethanol for at least 1 h at room temperature. RNAs were eliminated from fixed cells using 0.25 mg/ml of RNAse A overnight at 37°. Cells were subsequently washed twice using sodium citrate (50 mM, pH 7) and stained with a final SYTOX green concentration of 0.6 μM for a minimum of 1 h at room temperature in the dark. The volume of cells was adjusted to be around a cell concentration of less than 500 cells/μL. Five thousand cells for each sample were analyzed on a Guava easyCyte 8HT flow cytometer using a sample tray for 96-well microplates. Cells were excited with the blue laser at 488 nm and fluorescence was collected with a green fluorescence detection channel (peak at 512 nm). The distributions of the green fluorescence values were processed to find the two main density peaks, which correspond to the two cell populations, respectively, in G1 and G2 phases. The data were analyzed using R v3.4.159.

### Sporulation and dissection

The frozen stock of the Jean-Talon strain was streaked for single colonies onto a fresh YPD agar plate (1% yeast extract, 2% glucose, 2% peptone, 2% agar). Three independent colonies were picked, and the cells were patched on a solid sporulation medium (1% Potassium acetate, 0.1% Yeast extract, 0.05% Glucose, 0.01% sporulation dropout, 2% Agar). The sporulation dropout was composed of 0.0125 g/L Histidine, 0.0625 g/L Leucine, 0.0125 g/L Lysine and 0.0125 g/L Uracil. After seven days of incubation at room temperature, for each patch, a lump of cells was picked with a 200 µL micropipette tip and resuspended into 100 µL of a zymolyase solution (4 U/ml of Zymolyase, Zymolyase 20T, Bioshop Canada). After 20 min, cells were centrifuged for 20 sec at 16,100 g and the zymolyase solution was removed and replaced with 100 µL of a 1 M sorbitol solution. For each of the initial colonies, 24 tetrads were dissected on fresh YPD plates with a SporePlay dissection microscope (Singer Instruments, Somerset, UK). After five days of incubation at room temperature, plates were photographed, and fertility was determined as the number of visible colonies to the naked eye.

### Cobalt resistance assay

Three laboratory, two wild strains from oak tree bark in North America (Leducq *et al.* 2016) and two beer strains obtained from Lallemand (Table S1) were thawed from glycerol stocks on solid YPD petri dishes (30°, two days). Eight independent replicates from each strain were pre-cultured in 1 mL of YPD liquid cultures in 96 deep-well plates (2 ml) and incubated overnight at 30°. Cells were subsequently diluted to OD595 of 0.1 and grown to OD595 of 0.6 in the same conditions. A volume of 20 µL of these pre-cultures were grown in 96-well flat-bottomed culture plates in 180 µL of YPD media with the different concentrations of CoCl_2_ (0 mM, 2 mM, 4 mM, 6 mM, 8 mM and 10 mM), resulting in an initial OD595 of approximately 0.1. Incubation at 30° for 24 h was performed directly in three temperature-controlled spectrophotometers (Infinite 200 PRO, Tecan, Reading, UK) that read the OD595 at intervals of 15 min. The growth rate of each strain replicate was extracted from growth curves using R v3.6.1. The growth rate was computed as the 98^th^ percentile of the set of linear regression slopes fitted in five-timepoint wide overlapping sliding windows with a correlation coefficient r > 0.8.

### Short-read library construction and sequencing

Genomic DNA was extracted from an overnight culture derived from an isolated colony following standard protocols (QIAGEN DNAeasy, Hilden, Germany). The library was prepared with the Illumina Nextera kit (Illumina, San Diego, USA) following the manufacturer’s protocol and modifications described in (Baym *et al.* 2015). The library was sequenced with the 150 bp PE mode in a lane of HiSeqX (Illumina, San Diego, USA) at the Genome Quebec Innovation Center (Montréal, Canada). Genome-wide coverage reached 75X after duplicate reads removal.

### Long-read library construction and sequencing

DNA was extracted following a standard phenol-chloroform method from an overnight culture inoculated with an isolated colony of the Jean-Talon strain. PCR-free libraries for Oxford Nanopore Technologies (ONT) sequencing were prepared (in multiplex with other yeast strains) with kits SQK-LSK109 and EXP-NBD104 (Oxford Nanopore, Oxford, UK). Sequencing was performed on a FLO-MIN106 (revC) flowcell on a MinION sequencer (MIN-101B) driven by a MinIT computer (MNT-001) running the MinKNOW software v3.3.2. Basecalling was performed on the MinIT with guppy v3.0.3. Demultiplexing was performed using the guppy_basecaller utility v3.1.5.

### Genotyping of the Jean-Talon strain

Illumina reads were mapped onto the S288C *S. cerevisiae* reference genome vR64.2.1 using bwa mem v0.7.17 (Li 2013). Duplicated reads were tagged using picard tools v2.18 (http://broadinstitute.github.io/picard/). Genotypes were called with GATK v3.8 (DePristo *et al.* 2011) using HaplotypeCaller with an option -ERC BP_RESOLUTION and GenotypeGVCFs with an option -includeNonVariantSites and -ploidy 2. Single nucleotide polymorphisms (SNPs) were filtered with VariantFiltration module, excluding variants annotated with QualbyDepth < 2, MappingQuality < 40, MappingQualityRankSumTest < -12.5, FisherStrand > 60, StrandOddsRatio > 3 and ReadPosRankSum < -8.0. Additionally, genotypes with quality < 20 phreds (both GQ and RGQ) and coverage < 10 reads were masked. Indels were excluded.

### Combining Jean-Talon SNPs with other datasets

To combine variants of the Jean-Talon strain with the published yeast variants, VCF files with SNPs from Fay *et al.* 2019 (hereafter “Fay *et al.* dataset”), and Peter *et al.* 2018 (hereafter “1000 yeast dataset”) were downloaded. Only positions in the Jean-Talon strain present also in the Fay *et al.* and 1000 yeast datasets were retained using bcftools v1.9 (Li *et al.* 2009), after adjusting chromosome names. The two datasets were combined with the Jean-Talon VCF file separately using GATK v3.8 (DePristo *et al.* 2011) CombineVariants module with an option -genotypeMergeOptions UNIQUIFY. Multiallelic SNPs were removed from respective merged datasets using bcftools v1.9. Principal Component Analysis was performed using SNPrelate v1.18.1 package (Zheng *et al.* 2012) in R v3.6.1.

### Genotyping and comparison of beer strains

To find yeast strains that are genetically closest to the Jean-Talon strain, yeast genomes from genetic beer groups from four studies (Gallone *et al.* 2016; Gonçalves *et al.* 2016; Peter *et al.* 2018; Fay *et al.* 2019) were downloaded and mapped (Table S2). In total 319 strains were analyzed, including the Jean-Talon strain. Reads were trimmed for the common Illumina adapters with Trimmomatic v0.36 (Bolger *et al.* 2014), and mapped to the S288C *S. cerevisiae* genome using bwa mem v0.7.17 (Li 2013). Duplicate reads were tagged with picard tools v2.18. SNPs were called and filtered with GATK v4.1, as described above, but excluding filters, which are affected by single reads, such as FisherStrand and StrandOddsRatio. SNPs with less than 10% of missing data across all strains were retained. The SNPrelate v1.18.1 package (Zheng *et al.* 2012) in R v3.6.1 was used to calculate identity by state and identity by descent. Neighbor-joining tree was built using identity by state matrix with R package ape v5.3. Kinship coefficient matrix (identity by descent) was estimated with KING method of moment. To estimate nucleotide diversity and divergence between closely related strains, five beer strains closely related to the Jean-Talon strain (A.Muntons, A.S-33, BE005, CFI and CFN; A.Windson was not included due to large amount of missing data), three other beer strains from the Beer/baking group (CHK, CFP, A.T-58) and one strain from the Ale2 group (A.2565) were genotyped in all genomic positions using GATK v4.1, as described above but with -ploidy option 4 (except for CHK which is diploid). Genotypes passing all the filters were transferred on four (or two) reference genome sequences using seqtk v1.3 (https://github.com/lh3/seqtk), and other positions were marked as missing data. Coding sequences of 5713 single exon, non-overlapping genes were extracted and concatenated to generate multiple sequence alignments using bedtools v2.25 (Quinlan and Hall 2010) and custom Python v3.6.8 scripts (File S1). Summary statistics and number of synonymous sites were calculated using mstatspop v.0.1beta (https://github.com/CRAGENOMICA/mstatspop). The number of generations separating Jean-Talon and its close relatives was calculated by estimating what fraction of the total branch length to the common ancestor with S288C it constitutes (Green *et al.* 2006; Skoglund *et al.* 2011). To estimate fraction of the total length to the common ancestor with S288C, two patterns of synonymous variants were counted: variants that are shared between Jean-Talon and S288C, but not with the relative, and variants that are shared between relative and S288C, but not with the Jean-Talon. The counts were averaged and divided by the total number of synonymous sites with no missing data. Shared synonymous variants were identified using custom Python v3.6.8 script (File S1) after annotating variants in VCF file using SnpEff (Cingolani *et al.* 2012). Divergence time with S288C was estimated with molecular clock, assuming mutation rate 1.67E-10 per base per generation (Zhu *et al.* 2014), and considering only synonymous substitutions.

Copy number profiles were obtained in 250 bp non-overlapping windows in all strains with Control-FREEC v11.5 (Boeva *et al.* 2011, 2012). Strains with genome-wide coverage depth below 10X were excluded. First, ploidy of each strain was inferred using nQuire using reads with mapping quality > 30 phreds, and lrdmodel option (Weiß *et al.* 2018). However, some estimates of ploidy were not consistent with prior information, therefore instead of copy numbers inferred in Control-FREEC, normalized coverage estimates were used. Control-FREEC was run using options breakPointThreshold = 0.8, minExpectedGC = 0.35, maxExpectedGC = 0.55, telocentromeric = 7000 and window size set to 250 bp. Some strains showed nonuniform coverage across chromosomes with either increasing or decreasing coverage toward chromosome ends. Since this pattern can adversely affect loss and gain estimation additional calculations were done to identify and filter them out. In each strain a linear model was fitted, with x being a normalized distance from the midpoint (x = 0) to an end (x = 1.0) of a chromosome, and y being the binary logarithm of normalized coverage in 250 bp windows. Coverage was constrained between -2 and 2 to reduce noise. Strains with the slope below -0.15 and above 0.15 were filtered out (in total 102 strains). Additionally, 8 outlier strains with an exceptionally high number of detected gains and losses with mean length less than 50 kb were excluded. To compare depth of coverage in maltose metabolic process genes (GO:0000023), normalized coverage was averaged for windows overlapping each gene. The reference genome lacks some of the maltose genes, such as *MAL4* or *MAL6*, which are homologous to other *MAL* genes, and in case of their presence in the genome, could potentially affect read coverage of *MAL* genes. Although we cannot precisely estimate the number of copies of maltose metabolism genes, we can use our approach to roughly distinguish different categories of beer strains. To compare frequencies of copy number variants (CNVs) between genetic and environmental groups, we analyzed separately gains and losses of at least 10 kb, and with Wilcoxon Rank Sum Test p-value less than 0.05. Many strains are closely related to each other, therefore, to minimize the effect of relatedness in estimating frequencies of copy variants within groups, strains with kinship coefficient < 0.18 were randomly selected 20 times. Each time we made sure that at least one strain was present in each group. Mean frequency in each group was estimated for all CNVs present in the Beer/baking group. Only CNVs overlapping 75% of the Beer/baking CNV length were considered.

### Detecting introgression from Saccharomyces species

To detect potential gene flow between the Jean-Talon strain and other *Saccharomyces* species, competitive mapping was performed using SppIDER (last download 29/09/2019, (Langdon *et al.* 2018)). In the first analysis, Jean-Talon reads were mapped simultaneously to eight *Saccharomyces* species assemblies: *S. paradoxus* (ASM435296v1), *S. cerevisiae* (S288C vR64.2.1), *S. eubayanus* (SEUB3.0), *S. jurei* (SacJureiUoM1), *S. kudriavzevii* (ASM332763v1), *S. mikatae* (ASM16697v1), *S. uvarum* (ASM224264v1) and *S. arboricolus* (SacArb1.0). In the second analysis, reads were mapped to assemblies of six *S. paradoxus* lineages: *Ci* (ASM435303v1), *D2* (ASM435295v1), *A* (ASM435296v1), *D1* (ASM435294v1), *B* (ASM435310v1), *C* (ASM435309v1) and the genome assembly of *S. cerevisiae* (S288C vR64.2.1). All assemblies were masked using RepeatMasker v4.0.7 (http://www.repeatmasker.org) prior to the analysis.

### Assembly of the Jean-Talon genome

The Jean-Talon genome was assembled using the ONT dataset. We used wtdbg2 v2.5 (Ruan and Li 2019) with parameters -x ont -g 12m. The ONT reads were mapped against the draft assembly using minimap2 v2.17 (Li 2018) with parameter -x map-ont. The draft assembly was then polished using Nanopolish v0.11.1 (Loman *et al.* 2015) with parameter --min-candidate-frequency 0.1. Illumina reads were mapped against the signal-level polished assembly using bwa mem v0.7.16 (Li 2013) and the alignment was used to further polish the assembly using Pilon v1.22 (Walker *et al.* 2014). The polished assembly was aligned to the S288C reference genome from (Yue *et al.* 2017) using Mauve v2.4.0 (Darling *et al.* 2010), following which contigs were reordered to match reference chromosomes. Contig ctg6_pilon was manually split at the Ty2 junction as our structural analysis provided no support for the assembled translocation (see below). Visualizations of translocations were produced with the Mauve GUI.

### Simulation of translocations in the S288C genome

The S288C genome was used to simulate three reciprocal translocations. Our goal was to estimate the power of a split mapping approach to detect rearrangements occurring at full-length Ty retrotransposon loci, since those are large (∼6 kb) dispersed repeats which are expected to produce non-unique mappings. Using the genome annotations of (Yue *et al.* 2017) (coordinates are shown in parenthesis), two pairs of same-strand, full-length Ty1 elements were selected. Translocations were simulated between members of a pair using a custom Python v3.7.1 script. The first translocation is between a subtelomeric Ty1 on chromosome VIII (562107-568134) and a Ty1 on chromosome XIII (378473-384398). The second translocation is between Ty1s on chromosomes IV (1214433-1220350) and XIV (512688-518577). A third translocation with genic breakpoints was simulated between YER068W on chromosome V (293281-295044) and YJR010W on chromosome X (462156-463691). The rearranged assembly harboring the three translocations was used to simulate PacBio reads using PBSIM v1.0.4 (Ono *et al.* 2013) with parameters --data-type CLR --depth 600 --length-mean 3000 --length-sd 2300 and the default error model.

### Structural variants (SVs) analysis using long reads

ONT reads for Jean-Talon (this study), PacBio reads for A.2565, A.T-58 (Fay *et al.* 2019) and S288C (Yue *et al.* 2017) and simulated PacBio reads for S288C were filtered with SeqKit (Shen *et al.* 2016) to keep read lengths between 8 kb and 20 kb inclusively. The filtered reads were mapped on the S288C reference genome (Yue *et al.* 2017) using minimap2 v2.17 with parameters -x map-ont or -x map-pb. SVIM v1.1.1 (Heller and Vingron 2019) was used to call five classes of SVs (deletions, insertions, tandem or interspersed duplications, inversions) based on the long-read alignments. Since the coverage depth of the Jean-Talon library was higher (59X) than that of A.2565 (9X) and A.T-58 (12X), the Jean-Talon library was randomly subsampled to approximately 9X using seqtk (https://github.com/lh3/seqtk) to correct for potential coverage depth biases in the detection of SVs. SVs supported by a number of reads lower than 15% of the coverage depth were filtered out. For each strain, we derived the distribution of physical distances from an SV call to the closest SV call of the same class in each of the other strains. Using the distribution of distances between the two Jean-Talon datasets (59X and 9X coverage depth) as a reference, we used one-sided Mann-Whitney *U*-tests to determine which distributions were significantly shifted toward larger distances. Interspersed duplications and inversions were excluded from this analysis, as they comprised few or no calls across the datasets.

### Translocation analysis using split mapping

Long-read split mappings were used to search for translocations in the Jean-Talon, A.2565 and A.T-58 genomes compared to S288C. From the previously described long-read alignments, we extracted read IDs which had supplementary mappings and no secondary mapping using samtools v1.9 (Li *et al.* 2009). The alignments were filtered according to these read IDs using picard FilterSamReads v2.18.5 (http://broadinstitute.github.io/picard/) and subsequently analyzed using custom Python v3.7.1 scripts. Keeping only the split reads which map to exactly two different chromosomes, we binned them in 20 kb non-overlapping windows and represented read segment mappings with heatmaps. The length of the supporting reads was used to convert counts of supporting reads into approximate fraction of genome-wide coverage depth. Mappings of the S288C PacBio dataset against the reference S288C assembly allowed to identify artifactual signals arising from the split mapping approach. Mappings of the S288C simulated PacBio dataset against the reference S288C assembly allowed to compare the power of the split mapping approach to detect translocations at Ty and genic breakpoints.

### Translocation analysis using draft reassemblies

Two additional draft assemblies of the Jean-Talon were performed to investigate the presence of the translocation detected in the original wtdbg2 assembly. To maximize the quality of these two assemblies, filters were applied to use only reads longer than 8 kb. First, a draft assembly was produced using wtdbg2 v2.5 with parameters -p 0 -k 15 -AS 2 -s 0.05 -L 8000 -g 12m -X 120. Second, a draft assembly was produced using Canu v1.9 (Koren *et al.* 2017) with parameters -fast genomeSize = 12m minReadLength = 8000. The draft contigs produced by wtdbg2, the draft contigs and unitigs produced by Canu and the S288C PacBio reference assembly were aligned to the polished wtdbg2 assembly using the program nucmer v3.1 from the MUMmer 3.23 suite (Kurtz *et al.* 2004) with option --mum set.

### Data availability

Raw short and long (basecalled, demultiplexed) sequencing reads and the genome assembly are available at NCBI (PRJNA604588). Code to analyze the data and plot the figures is available at https://github.com/Landrylab/Fijarczyk2020_JeanTalon. Description of strains used in the growth assay is in Table S1. Table S2 contains metadata of strains mapped and genotyped in this study. Estimates of divergence time between Jean-Talon and related beer strains are in Table S3. File S1 contains code used to analyze the data and plot the figures. VCF file with SNPs of the Jean-Talon and strains from the Fay *et al.* dataset is in File S2. VCF file with SNPs of the Jean-Talon and strains from the 1000 yeast dataset is in File S3. VCF file with SNPs of all yeast strains mapped and genotyped in this study is in File S4. VCF file with polyploid genotypes of strains related to the Jean-Talon is in File S5. File S6 contains estimates of normalized coverage in 250 bp windows for all strains mapped in this study. File S7 contains gain and loss variants for all strains mapped in this study. Figure S1 shows pictures of colonies and cells of the Jean-Talon strain. Figure S2 shows lack of evidence for gene flow between Jean-Talon and other *Saccharomyces* species. Figure S3 shows results of CNV analyses in yeast strains. Figure S4 shows translocations from genome assembly and simulations. Figure S5 shows read length distribution of long read datasets used in the study. Figure S6 shows results of translocation detection using split mapping. Figure S7 shows results of translocation detection using draft assemblies. Supplemental material available at figshare: https://doi.org/10.25387/g3.12324308.

## Results and Discussion

The colonies of the Jean-Talon strain grown on YPD medium are creamy-beige, round, with convex elevation and matt finish, with quite smooth and creamy surface. The diameter of most colonies is around 3 mm (Figure S1). Under the microscope the cells look round, medium and uniform in size and shape and are arranged in clusters (Figure S1), consistent with being *S. cerevisiae*.

### The Jean-Talon strain is a tetraploid strain and is largely sterile

**Figure 1:**
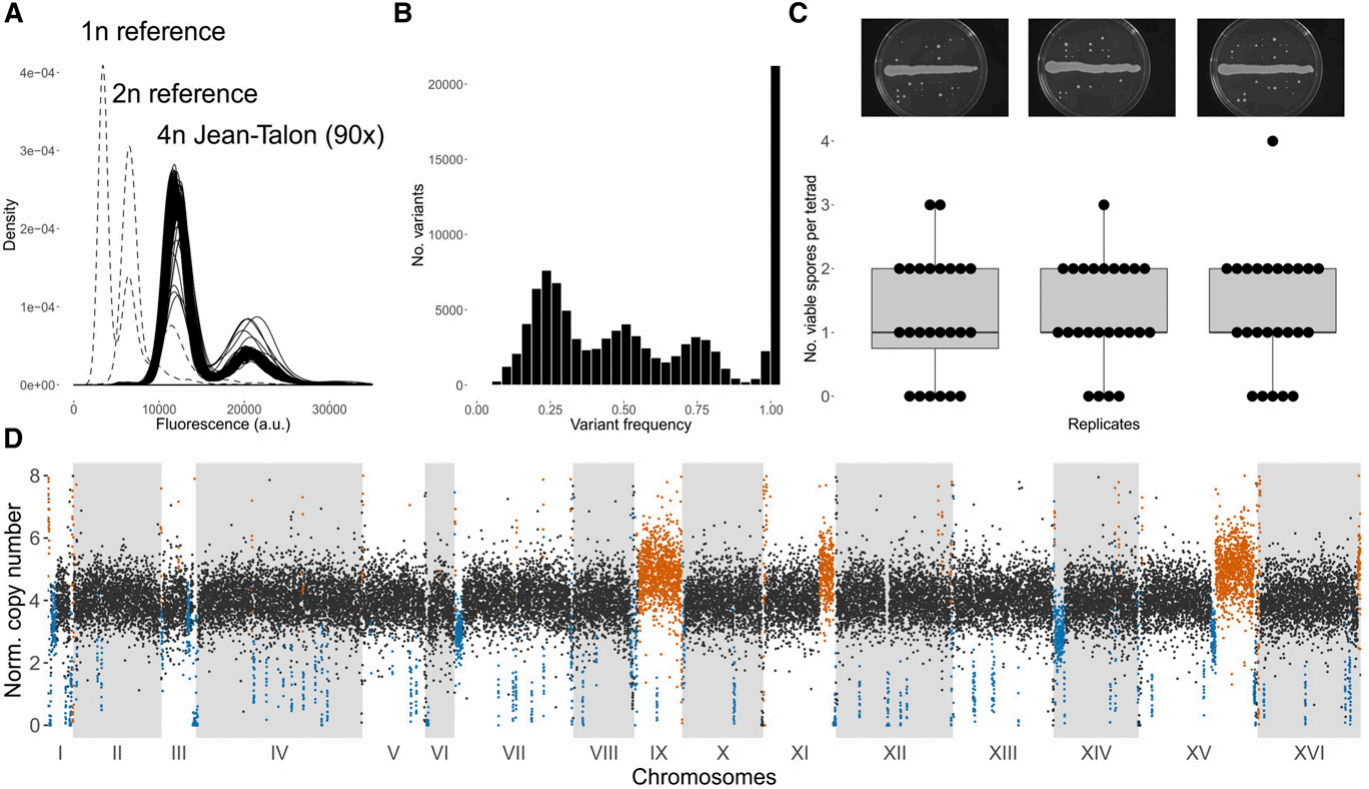
The Jean-Talon strain is a tetraploid with reduced spore viability. (A) DNA staining and fluorescence measured for 90 colonies of the Jean-Talon strain shows peaks around the expected ploidy of 4*n*. (B) SNP frequency distribution in the Jean-Talon genome mapped to *S. cerevisiae* S288C. (C) Boxplots and numbers of viable spores per tetrad found in three biological replicates of sporulated cultures. Pictures of the spore dissection plates for the corresponding replicates are shown above. (D) Copy number profile up to 8*n* of the Jean-Talon genome in 250 bp windows. Black windows correspond to the expected ploidy 4*n*, orange windows correspond to copy number gains and blue windows to copy number losses. Long stretches of copy number gains and losses correspond to ploidy of 5*n* and 3*n*, respectively. Code to generate the figures is in File S1.

We first examined the ploidy and the ability of the Jean-Talon strain to sporulate. DNA staining of 90 isolated colonies shows that it is a tetraploid (Figure 1A). The frequency distribution of single nucleotide polymorphisms (SNPs) mapped to the S288C genome shows peaks around frequencies of 0.25, 0.5 and 0.75 also indicating tetraploidy (Figure 1B). A recent study of the genomes of 1011 *S. cerevisiae* strains revealed that most natural isolates were diploid (Peter *et al.* 2018). However, approximately 11.5% of isolates were polyploid (3–5*n*) and those were enriched in specific subpopulations such as the beer, mixed-origin and African palm wine clades, which strongly suggests that some human-related environments have had an effect on the ploidy level (Peter *et al.*, 2018). Similar results from Gallone *et al.* 2016 and Gonçalves *et al.* 2016 showed that multiple populations of beer strains had high rates of tetraploidy. Although spontaneous yeast tetraploids are usually fertile (Charron *et al.* 2019), the Jean-Talon strain shows about 30% spore viability (Figure 1C), which is close to the average spore viability observed for beers from the genetic groups Beer2, Mixed and Mosaic in (Gallone *et al.* 2016). The Jean-Talon is therefore typical of beer strains with respect to ploidy and fertility.

Several long-range copy number gains and losses were observed in the genome, including the presence of five copies of chromosome IX, similar copy number changes at the ends of chromosomes XI and XV, and three copies at the beginning of chromosomes I, VII, XIV and middle of chromosome XV (Figure 1D). The aneuploidies and CNVs are typical of what is observed for industrial yeast strains (Gallone *et al.* 2016), but are rare in species that have not been domesticated (Leducq *et al.* 2016; Yue *et al.* 2017).

### The Jean-Talon strain belongs to the Beer/baking beer group

**Figure 2:**
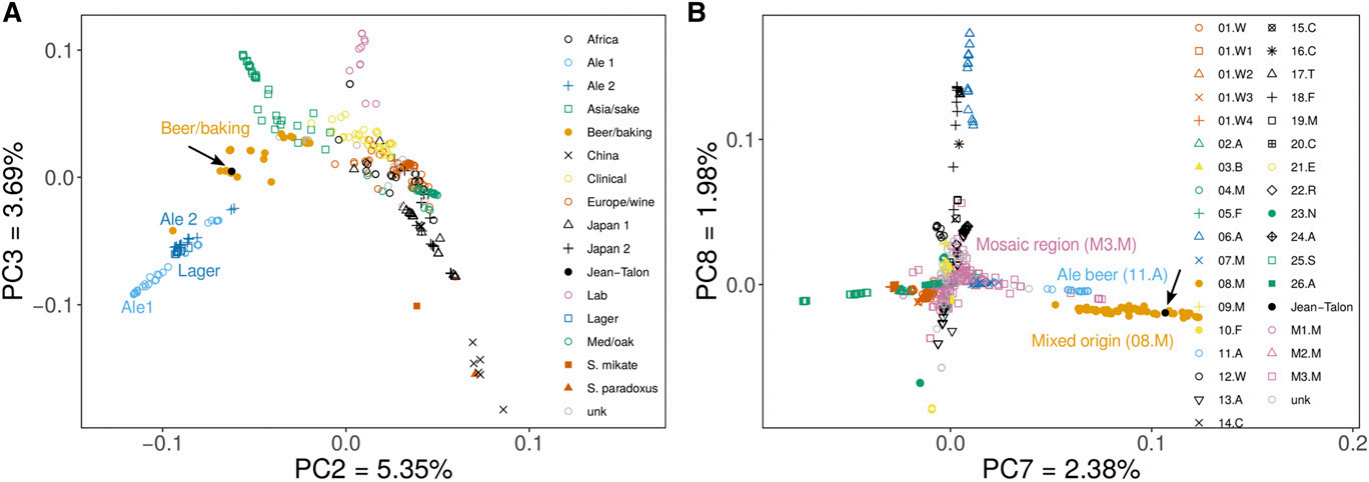
SNPs suggest that Jean-Talon belongs to the Beer/baking beer group. (A) PCA with 257,116 biallelic SNPs from 402 yeast strains from Fay *et al.* dataset groups Jean-Talon (black filled dot) within the Beer/baking group. (B) PCA with 314,366 biallelic SNPs from 1000 yeast dataset (1012 yeast strains) groups Jean-Talon within the Mixed origin group. Position of the Jean-Talon strain is indicated with an arrow. “unk” refers to unclustered strains. Code to generate the figures is in File S1. The underlying data containing SNPs is in Files S2-S3.

To find out to which genetic group Jean-Talon belongs to, we combined SNPs of the Jean-Talon with two yeast datasets: 401 strains from Fay *et al.* (Fay *et al.* 2019), and 1011 strains from 1000 yeast (Peter *et al.* 2018). Principal Component Analysis (PCA) on the Fay *et al.* dataset shows that Jean-Talon groups with the beer strains from the Beer/baking group according to PC2 and PC3 (Figure 2A), whereas PCA on the 1000 yeast dataset shows Jean-Talon grouping with the corresponding Mixed origin group, according to PC7 and PC8 (Figure 2B). The Mixed origin and Beer/baking groups comprise strains obtained from bakeries, breweries, as well as strains found in nature. Because the Jean-Talon strain was isolated from the environment, it may have mixed with other species, particularly *S. paradoxus*, which is found in Northern parts of North America (Charron *et al.* 2014) and with which it was shown to hybridize in different contexts (Barbosa *et al.* 2016). We did not detect gene-flow between the Jean-Talon and other *Saccharomyces* species using competitive mapping (Figure S2).

**Figure 3:**
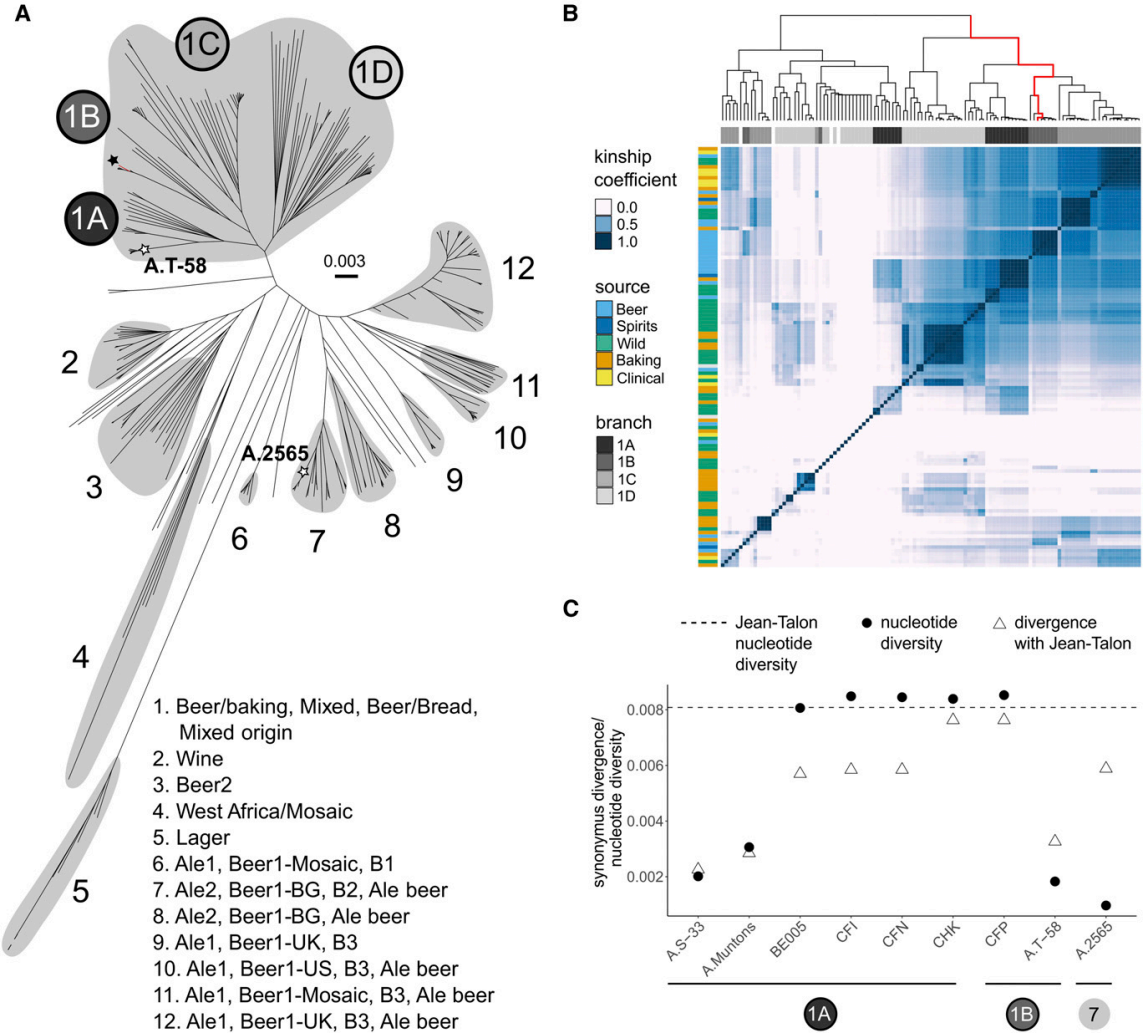
Jean-Talon is closely related to several commercial beer strains from the Beer/baking group. (A) Neighbor joining tree based on genome-wide genotype dissimilarity matrix for beer yeast strains from the four studies, Fay *et al.* 2019 (Ale1, Ale2, Lager, Beer/baking), Gallone *et al.* 2016 (Beer1*, Beer2, Mixed, Wine, West Africa, *Mosaic), Gonçalves *et al.* 2016 (B1, B2, B3, Beer/Bread) and Peter *et al.* 2018 (Mixed origin, Ale beer). Consecutive numbers describe group affiliation of larger branches according to different studies. Note that one longest branch of the lager strain was cut to fit in the figure (dotted lines). The strain of Jean-Talon within the Beer/baking group is depicted with a black star, white stars depict the position of two beer strains with long-read sequencing data. (B) Heatmap of kinship coefficients estimated for all pairs of Beer/baking strains with 131,808 genome-wide SNPs. The Jean-Talon strain (red line on a dendrogram) has a kinship coefficient above 93% with six beer strains: CFI, CFN, BE005, A.Muntons, A.S-33, and A.Windson. (C) Nucleotide diversity in the Jean-Talon strain is higher than divergence between most closely related beer strains, suggesting that many heterozygous variants are shared between the strains. Circles with numbers depict tree branches from (A). Code to generate the figures is in File S1. The underlying data containing SNPs and all genotypes is in Files S4-S5.

To further investigate the Jean-Talon strain and identify the most closely related beer strains, we mapped the reads of 318 strains from four different studies, which include major beer groups (Table S2) (Gallone *et al.* 2016; Gonçalves *et al.* 2016; Peter *et al.* 2018; Fay *et al.* 2019). Based on genotype similarity, the Jean-Talon strain is located on a branch composed of commercial beer strains (Figure 3A). The strain is nearly identical (kinship coefficient between 93% and 97%) to the six other beer strains (Figure 3B), which include commercially available yeast strains: Safale S-33 (isolates A.S-33 and CFN), Windsor (isolates A.Windson and CFI), Muntons (isolate A.Muntons) and isolate BE005 extracted from Belgian ale, all of which are used to produce English or Belgium-style ales (Table S2). Estimates of synonymous heterozygosity and pairwise divergence between these strains support the finding that most segregating variants in Jean-Talon are shared with other strains (Figure 3C). Using the molecular clock, the time of divergence between the Jean-Talon and the S288C reference genome is about 14 M generations. Assuming constant mutation rate, the mean time of divergence of Jean-Talon from its closest relatives is equal on average to a fraction of 0.0024 of divergence time with S288C, which translates to around 18,894-43,935 generations, depending on the strain (Table S3). The suggested number of generations per year in domesticated and lab yeast ranges from 150 (Gallone *et al.* 2016) to 2920 per year (Fay and Benavides 2005), suggesting the split could have occurred as recently as 6 years ago and as late as 126 years ago for the least diverged strain (Table S3). However, the growth of Jean-Talon in the vaults could have been different from other beer strains. Generation time can be overestimated if breweries use the same yeast stock for each batch of fermentation, instead of continuously transferring yeast from one fermentation to the next. Although we do not know for how long the strain had been active or dormant within the vaults, a relatively small number of generations suggests that the split with other beer strains did not occur long ago. It is likely that the strain was used in the Boswell brewery which was still active in the 60s of the 20^th^ century. Strains related to Jean-Talon were sampled from commercial ales, brewed from general purpose common yeast strains used for brewing different styles of beer, therefore they could have originated in the large commercial brewery.

### Distinct structural and phenotypic variation of the Jean-Talon strain

**Figure 4:**
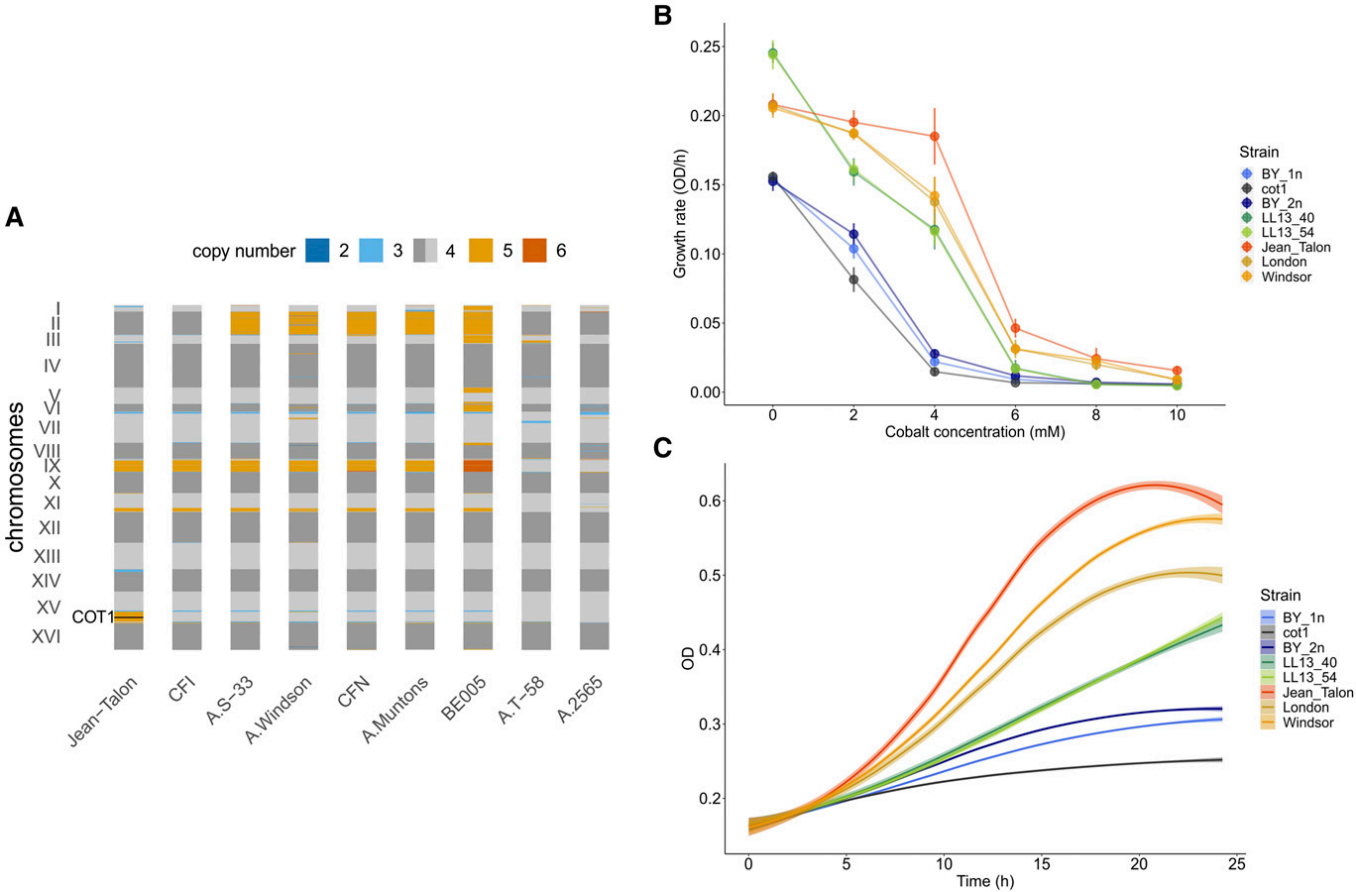
The Jean-Talon strain has some unique genomic and phenotypic traits compared to other related beer strains. (A) Copy number profiles for regions of at least 10 kb, in which > 90% of 250 bp windows have a given copy number. Jean-Talon has five copies of a 350 kb long fragment on chromosome XV which is present in only four copies in other related beer strains. This copy gain includes the *COT1* gene (black line), whose increased expression confers higher resistance to cobalt in yeast (Conklin *et al.* 1992). (B) Growth rates of *S. cerevisiae* laboratory, natural and beer strains. Jean-Talon shows the highest tolerance to all concentrations of cobalt. The laboratory strains are BY haploid (BY4741) and diploid (BY4743) strains (BY-1n and BY-2n on the figure) and as a control the haploid BY deleted for the *COT1* gene (cot1). The two natural strains, LL13_40 and LL13_54 are diploid wild strains isolated from oak tree bark in North America (Leducq *et al.* 2016). Windsor is a tetraploid beer stain closely related to Jean-Talon (two isolates of this strain are on panel A named as A.Windson and CFI). London is likely a tetraploid beer strain from the Ale1 genetic group. (C) Growth curves of *S. cerevisiae* laboratory, natural and beer strains in 6 mM cobalt measured over 24 h. Code and data to generate the figures is in File S1. The underlying raw data containing CNVs is in Files S6-S7.

We examined the copy number of genes that have been associated with adaptation of beer strains to the brewing environment, for instance maltose metabolism genes. Hierarchical clustering of the beer strains based on the coverage depth in genes involved in maltose metabolic process groups Jean-Talon with other English and Belgian ales similarly to SNPs (Figure S3). The profiles of large (> 10 kb) CNVs across the genomes of the Jean-Talon related strains show multiple, mostly shared aneuploidies, supporting their recent divergence (Figure 4A). Most of these CNVs are also shared with other beer and bakery strains (Figure S3). We also observe that some of these CNVs are frequent within the beer strains from other genetic groups, but are lacking or are very rare in bakery strains in the Beer/baking group (*i.e.*, copy gain of the chromosome III or copy loss at the beginning of chromosome VII, Figure S3), suggesting that they could be related to the brewing process. In particular we find that a short (around 15 kb) copy loss in the beginning of chromosome VII, which can be also found in Ale1 or Ale2 genetic groups, includes one alcohol dehydrogenase gene (*ADH4*), with potential role in the fermentation process.

In contrast to closely related beer strains, Jean-Talon carries an additional copy of a 350 kb region on chromosome XV (Figure 3A). A copy gain including this region is rare among all other analyzed strains (frequency 0.064). This copy variant includes*COT1*, a gene that is a major player in yeast resistance to cobalt, which leads to higher cobalt resistance when increased in copy number (Conklin *et al.* 1992). Cobalt has been used as a foam stabilizer in the brewing process in Québec City until 1968 (Morin and Daniel 1967). This CNV could therefore represent adaptation to this condition. To assess whether Jean-Talon strain has a higher resistance to cobalt, we compared its growth rate at different levels of cobalt concentrations to some laboratory, wild and other closely related beer strains (Table S1) that do not harbor the additional copy of *COT1* gene. We also included the *COT1* deletion strain as control. Our results confirm that Jean-Talon strain is significantly more resistant to higher concentrations of cobalt than all the other tested strains (Figure 4B, C).

**Figure 5:**
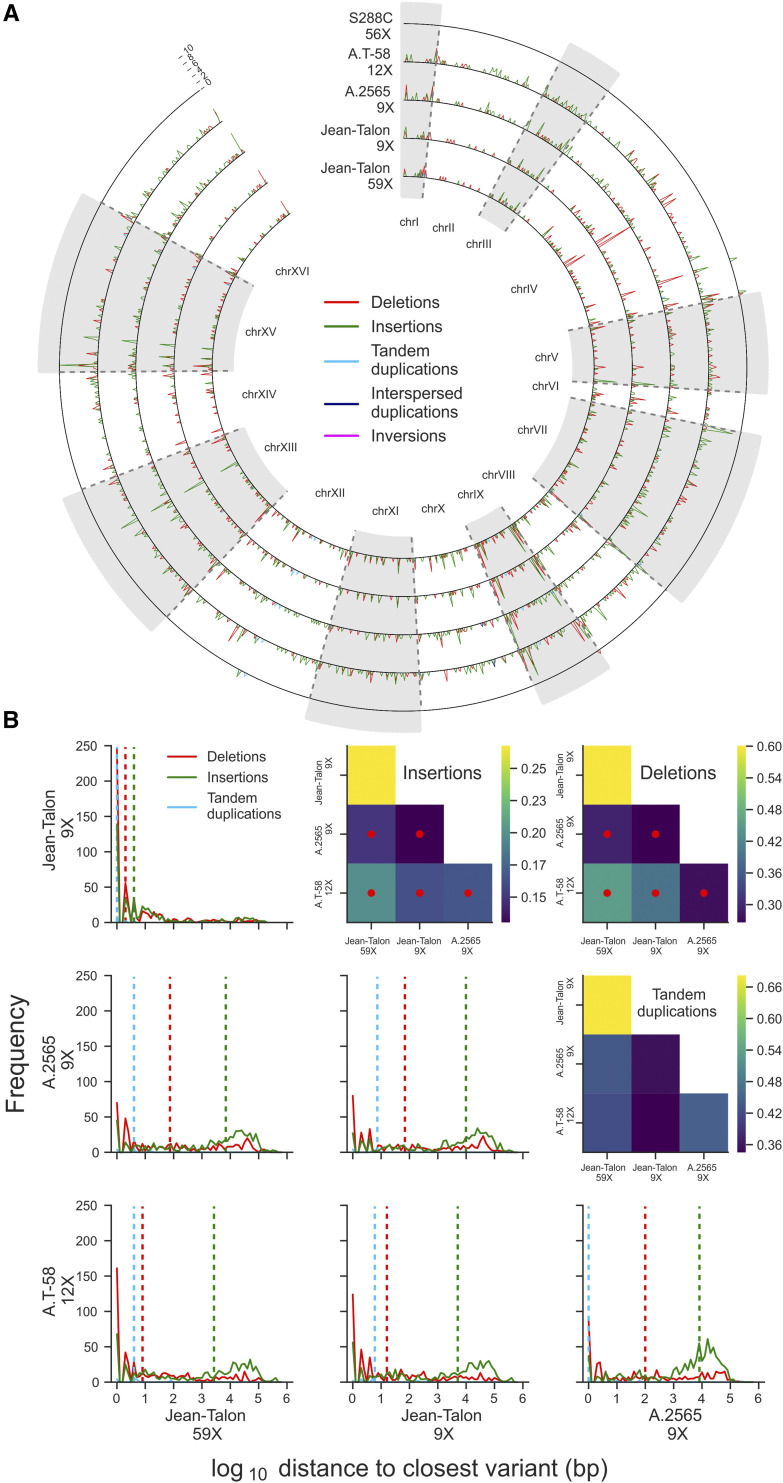
Structural variation (SV) in the genome of the Jean-Talon strain. (A) SVs compared to S288C for the Jean-Talon strain (complete and subsampled datasets), and two beer strains with available long read datasets, one from the Beer/baking group (A.T-58) and one from the Ale2 group (A.2565). SV density in non-overlapping 10 kb windows is plotted. (B) Physical proximity of SV calls between strains. Distributions of physical distance to the closest same-class SV call in the mate strain is shown for each pair of strains. Dotted vertical lines correspond to medians. Heatmaps show the results of two-sided Mann-Whitney *U*-tests for each distribution compared to the (Jean-Talon 9X: Jean-Talon 59X) reference pair. Color maps show ratios of U statistics to the reference, while red dots indicate distributions significantly right-shifted compared to the reference (p-values < 0.05, Mann-Whitney *U*-tests, FDR corrected). Data and code to generate the figures is in File S1.

We also called five classes of structural variants (SVs) based on mappings of long reads to the S288C reference genome (Figure 5A). Distributions of physical proximity of SV calls between strains show that Jean-Talon is closer to a strain from the Beer/baking group (A.T-58), rather than to a strain from the Ale2 group (A.2565, Figure 5B). Despite this relatedness, the Jean-Talon strain exhibits a distinct pattern of SVs as it is significantly different from both beer strains for the most abundant classes of SVs (insertions and deletions, Figure 5B).

We assembled the Jean-Talon genome using our Oxford Nanopore dataset and detected a translocation between chromosomes II and XI (Figure S4), whose breakpoint maps to a full-length Ty2 retrotransposon. We used split mappings of long reads to investigate translocations in the Jean-Talon, A.T-58 and A.2565 strains compared to S288C (Figure S5). Although we found this method has reduced power to detect translocations at Ty loci compared to genic breakpoints, we find no evidence for a translocation between chromosomes II and XI in Jean-Talon (Figure S6). Using an alternative assembler and excluding reads shorter than 8 kb yielded collinear draft assemblies, with single contigs for chromosome II and the right arm of chromosome XI (Figure S7, Files S1). However, high confidence contigs from the alternative assembler show that the translocation breakpoint is fragmented in a way that is consistent with misassembly of a full-length Ty2. The most likely explanations are that this translocation is either an assembly artifact or a true rearrangement present on a minority of haplotypes, making it hard to detect by our assembly-free method. Thus, the genomes of Jean-Talon and S288C appear to be largely collinear.

## Conclusion

The yeast Jean-Talon strain was isolated from an archeological site in the old part of Québec City where the first brewery was founded in the 17^th^ century. The strain was isolated from the vaults of the Second Intendant’s Palace that was built in the 18^th^ century and occupied by the Boswell brewery starting in the 19^th^ century. The Jean-Talon strain is a strain of *Saccharomyces cerevisiae*, which is not found naturally in this part of North America (Charron *et al.* 2014). The strain is very closely related to other strains used in industrial brewing, suggesting that it derived recently from other industrial beer strains. The high tolerance to cobalt suggests adaptation to cobalt usage in brewing, which directly links the Jean-Talon strain to more recent brewery activities on the site in the previous century.

## References

[bib1] Barbosa R., Almeida P., Safar S. V. B., Santos R. O., Morais P. B., 2016 Evidence of natural hybridization in Brazilian wild lineages of Saccharomyces cerevisiae. Genome Biol. Evol. 8: 317–329. 10.1093/gbe/evv263 26782936 PMC4779607

[bib2] Baym M., Kryazhimskiy S., Lieberman T. D., Chung H., Desai M. M., 2015 Inexpensive multiplexed library preparation for megabase-sized genomes. PLoS One 10: e0128036 10.1371/journal.pone.0128036 26000737 PMC4441430

[bib3] Boeva V., Popova T., Bleakley K., Chiche P., Cappo J., 2012 Control-FREEC: a tool for assessing copy number and allelic content using next-generation sequencing data. Bioinformatics 28: 423–425. 10.1093/bioinformatics/btr670 22155870 PMC3268243

[bib4] Boeva V., Zinovyev A., Bleakley K., Vert J.-P., Janoueix-Lerosey I., 2011 Control-free calling of copy number alterations in deep-sequencing data using GC-content normalization. Bioinformatics 27: 268–269. 10.1093/bioinformatics/btq635 21081509 PMC3018818

[bib5] Bolger A. M., Lohse M., and Usadel B., 2014 Trimmomatic: a flexible trimmer for Illumina sequence data. Bioinformatics 30: 2114–2120. 10.1093/bioinformatics/btu170 24695404 PMC4103590

[bib6] Cavalieri D., McGovern P. E., Hartl D. L., Mortimer R., and Polsinelli M., 2003 Evidence for S. cerevisiae fermentation in ancient wine. J. Mol. Evol. 57: S226–S232. 10.1007/s00239-003-0031-2 15008419

[bib7] Charron G., Leducq J.-B., Bertin C., Dubé A. K., and Landry C. R., 2014 Exploring the northern limit of the distribution of Saccharomyces cerevisiae and Saccharomyces paradoxus in North America. FEMS Yeast Res. 14: 281–288. 10.1111/1567-1364.12100 24119009

[bib8] Charron G., Marsit S., Hénault M., Martin H., and Landry C. R., 2019 Spontaneous whole-genome duplication restores fertility in interspecific hybrids. Nat. Commun. 10: 4126 10.1038/s41467-019-12041-8 31511504 PMC6739354

[bib9] Cingolani P., Platts A., Wang L. L., Coon M., Nguyen T., 2012 A program for annotating and predicting the effects of single nucleotide polymorphisms, SnpEff: SNPs in the genome of Drosophila melanogaster strain w1118; iso-2; iso-3. Fly (Austin) 6: 80–92. 10.4161/fly.19695 22728672 PMC3679285

[bib10] Conklin D. S., McMaster J. A., Culbertson M. R., and Kung C., 1992 COT1, a gene involved in cobalt accumulation in Saccharomyces cerevisiae. Mol. Cell. Biol. 12: 3678–3688. 10.1128/MCB.12.9.3678 1508175 PMC360222

[bib11] Darling A. E., Mau B., and Perna N. T., 2010 progressiveMauve: multiple genome alignment with gene gain, loss and rearrangement. PLoS One 5: e11147 10.1371/journal.pone.0011147 20593022 PMC2892488

[bib12] DePristo M. A., Banks E., Poplin R., Garimella K. V., Maguire J. R., 2011 A framework for variation discovery and genotyping using next-generation DNA sequencing data. Nat. Genet. 43: 491–498. 10.1038/ng.806 21478889 PMC3083463

[bib13] Fay J. C., and Benavides J. A., 2005 Evidence for domesticated and wild populations of Saccharomyces cerevisiae. PLoS Genet. 1: e5 10.1371/journal.pgen.0010005 PMC1183524 16103919

[bib14] Fay J. C., Liu P., Ong G. T., Dunham M. J., Cromie G. A., 2019 A polyploid admixed origin of beer yeasts derived from European and Asian wine populations. PLoS Biol. 17: e3000147 10.1371/journal.pbio.3000147 30835725 PMC6400334

[bib15] Fiset R., 2001 Brasseries et distilleries à Québec (1620-1900): profil d'archéologie industrielle [Doctoral dissertation]: Université Laval.

[bib16] Gallone B., Steensels J., Prahl T., Soriaga L., Saels V., 2016 Domestication and Divergence of Saccharomyces cerevisiae Beer Yeasts. Cell 166: 1397–1410.e16. 10.1016/j.cell.2016.08.020 27610566 PMC5018251

[bib17] Gerstein A. C., Chun H.-J. E., Grant A., and Otto S. P., 2006 Genomic convergence toward diploidy in Saccharomyces cerevisiae. PLoS Genet. 2: e145 10.1371/journal.pgen.0020145 17002497 PMC1570378

[bib18] Gonçalves M., Pontes A., Almeida P., Barbosa R., Serra M., 2016 Distinct Domestication Trajectories in Top-Fermenting Beer Yeasts and Wine Yeasts. Curr. Biol. 26: 2750–2761. 10.1016/j.cub.2016.08.040 27720622

[bib19] Green R. E., Krause J., Ptak S. E., Briggs A. W., Ronan M. T., 2006 Analysis of one million base pairs of Neanderthal DNA. Nature 444: 330–336. 10.1038/nature05336 17108958

[bib20] Guimont J., 1987 Le site du Premier palais de l'intendant à Québec (Ce Et 30): rapport préliminaire de la quatrième campagne de fouilles (1985) [Master's thesis]: Université Laval.

[bib21] Heller D., and Vingron M., 2019 SVIM: structural variant identification using mapped long reads. Bioinformatics 35: 2907–2915. 10.1093/bioinformatics/btz041 30668829 PMC6735718

[bib22] Koren S., Walenz B. P., Berlin K., Miller J. R., Bergman N. H., 2017 Canu: scalable and accurate long-read assembly via adaptive k-mer weighting and repeat separation. Genome Res. 27: 722–736. 10.1101/gr.215087.116 28298431 PMC5411767

[bib23] Kurtz S., Phillippy A., Delcher A. L., Smoot M., Shumway M., 2004 Versatile and open software for comparing large genomes. Genome Biol. 5: R12 10.1186/gb-2004-5-2-r12 14759262 PMC395750

[bib24] Langdon Q. K., Peris D., Kyle B., and Hittinger C. T., 2018 sppIDer: A Species Identification Tool to Investigate Hybrid Genomes with High-Throughput Sequencing. Mol. Biol. Evol. 35: 2835–2849. 30184140 10.1093/molbev/msy166 PMC6231485

[bib25] Leducq J.-B., Nielly-Thibault L., Charron G., Eberlein C., Verta J.-P., 2016 Speciation driven by hybridization and chromosomal plasticity in a wild yeast. Nat. Microbiol. 1: 15003 10.1038/nmicrobiol.2015.3 27571751

[bib26] Li H., 2013 Aligning sequence reads, clone sequences and assembly contigs with BWA-MEM. arXiv:1303.3997 [q-bio.GN] (Preprint posted May 16, 2013).

[bib27] Li H., 2018 Minimap2: pairwise alignment for nucleotide sequences. Bioinformatics 34: 3094–3100. 10.1093/bioinformatics/bty191 29750242 PMC6137996

[bib28] Li H., Handsaker B., Wysoker A., Fennell T., Ruan J., 2009 The Sequence Alignment/Map format and SAMtools. Bioinformatics 25: 2078–2079. 10.1093/bioinformatics/btp352 19505943 PMC2723002

[bib29] Loman N. J., Quick J., and Simpson J. T., 2015 A complete bacterial genome assembled de novo using only nanopore sequencing data. Nat. Methods 12: 733–735. 10.1038/nmeth.3444 26076426

[bib30] Ludlow C. L., Cromie G. A., Garmendia-Torres C., Sirr A., Hays M., 2016 Independent Origins of Yeast Associated with Coffee and Cacao Fermentation. Curr. Biol. 26: 965–971. 10.1016/j.cub.2016.02.012 27020745 PMC4821677

[bib31] Marsit S., Leducq J.-B., Durand É., Marchant A., Filteau M., 2017 Evolutionary biology through the lens of budding yeast comparative genomics. Nat. Rev. Genet. 18: 581–598. 10.1038/nrg.2017.49 28714481

[bib32] McGovern P. E., Zhang J., Tang J., Zhang Z., Hall G. R., 2004 Fermented beverages of pre- and proto-historic China. Proc. Natl. Acad. Sci. USA 101: 17593–17598. 10.1073/pnas.0407921102 15590771 PMC539767

[bib33] Michel R. H., McGovern P. E., and Badler V. R., 1992 Chemical evidence for ancient beer. Nature 360: 24 10.1038/360024b0

[bib34] Morin Y., and Daniel P., 1967 Quebec beer-drinkers’ cardiomyopathy: etiological considerations. Can. Med. Assoc. J. 97: 926–928. 6051264 PMC1923410

[bib35] Moussette, M., 1992 La bière à l’époque de Jean-Talon. Cap-aux-Diamants 28: 18–20.

[bib36] Moussette, M., 1994 *Le site du Palais de l’intendant à Québec: genèse et structuration d’un lieu urbain*. Les éditions du Septentrion, Québec.

[bib37] Moussette M., 1996 The site of the intendant’s palace in Québec City: The changing meaning of an urban space. Hist. Archaeol. 30: 8–21. 10.1007/BF03373585

[bib38] Ono Y., Asai K., and Hamada M., 2013 PBSIM: PacBio reads simulator–toward accurate genome assembly. Bioinformatics 29: 119–121. 10.1093/bioinformatics/bts649 23129296

[bib39] Peter J., De Chiara M., Friedrich A., Yue J.-X., Pflieger D., 2018 Genome evolution across 1,011 Saccharomyces cerevisiae isolates. Nature 556: 339–344. 10.1038/s41586-018-0030-5 29643504 PMC6784862

[bib40] Quinlan A. R., and Hall I. M., 2010 BEDTools: a flexible suite of utilities for comparing genomic features. Bioinformatics 26: 841–842. 10.1093/bioinformatics/btq033 20110278 PMC2832824

[bib41] Ruan J., and Li H., 2020 Fast and accurate long-read assembly with wtdbg2. Nat. Methods 17: 155–158. 31819265 10.1038/s41592-019-0669-3 PMC7004874

[bib42] Shen W., Le S., Li Y., and Hu F., 2016 SeqKit: A Cross-Platform and Ultrafast Toolkit for FASTA/Q File Manipulation. PLoS One 11: e0163962 10.1371/journal.pone.0163962 27706213 PMC5051824

[bib43] Skoglund P., Götherström A., and Jakobsson M., 2011 Estimation of population divergence times from non-overlapping genomic sequences: examples from dogs and wolves. Mol. Biol. Evol. 28: 1505–1517. 10.1093/molbev/msq342 21177316

[bib44] Walker B. J., Abeel T., Shea T., Priest M., Abouelliel A., 2014 Pilon: an integrated tool for comprehensive microbial variant detection and genome assembly improvement. PLoS One 9: e112963 10.1371/journal.pone.0112963 25409509 PMC4237348

[bib45] Weiß C. L., Pais M., Cano L. M., Kamoun S., and Burbano H. A., 2018 nQuire: a statistical framework for ploidy estimation using next generation sequencing. BMC Bioinformatics 19: 122 10.1186/s12859-018-2128-z 29618319 PMC5885312

[bib46] Yue J.-X., Li J., Aigrain L., Hallin J., Persson K., 2017 Contrasting evolutionary genome dynamics between domesticated and wild yeasts. Nat. Genet. 49: 913–924. 10.1038/ng.3847 28416820 PMC5446901

[bib47] Zheng X., Levine D., Shen J., Gogarten S. M., Laurie C., 2012 A high-performance computing toolset for relatedness and principal component analysis of SNP data. Bioinformatics 28: 3326–3328. 10.1093/bioinformatics/bts606 23060615 PMC3519454

[bib48] Zhu Y. O., Siegal M. L., Hall D. W., and Petrov D. A., 2014 Precise estimates of mutation rate and spectrum in yeast. Proc. Natl. Acad. Sci. USA 111: E2310–E2318. 10.1073/pnas.1323011111 24847077 PMC4050626

